# Supplementary material for: Growing up with interfering neighbours: the influence of time of learning and vocabulary knowledge on written word learning in children
Source: R Soc Open Sci. 2020 Mar 25;7(3):191597. doi: 10.1098/rsos.191597 (PMC7137956; doi:10.1098/rsos.191597)
Supplement: Experimental stimuli [file rsos191597supp1.docx]

| **List 1** |  |  | **List 2** |  |  |
| --- | --- | --- | --- | --- | --- |
| **Base word** | **Novel word** | **Type** | **Base word** | **Novel word** | **Type** |
| ANCHOR | AMCHOR | Man-made | ARMPIT | ARFPIT | Natural |
| BANANA | BANARA | Natural | BISCUIT | BISCULT | Man-made |
| CARAVAN | CAGAVAN | Man-made | COCONUT | COCOSUT | Natural |
| FOSSIL | FASSIL | Natural | FLANNEL | FRANNEL | Man-made |
| GUITAR | GUITUR | Man-made | GARLIC | GARNIC | Natural |
| HAMSTER | HAGSTER | Natural | HELMET | HETMET | Man-made |
| MUSEUM | MUTEUM | Man-made | MEADOW | MEAROW | Natural |
| NEEDLE | NEEZLE | Man-made | NAPKIN | NASKIN | Man-made |
| RABBIT | ROBBIT | Natural | RAINBOW | RAINTOW | Natural |
| VOLCANO | VOPCANO | Natural | VIOLIN | VIODIN | Man-made |
| LANTERN | LANPERN | Man-made | LETTUCE | LETTUTE | Natural |
| POTATO | PORATO | Natural | PENCIL | PUNCIL | Man-made |
| SPIDER | SPIMER | Natural | SLEEVE | SPEEVE | Man-made |
| TEAPOT | TEAWOT | Man-made | TOMATO | TORATO | Natural |
