# Supplementary material for: Growing up with interfering neighbours: the influence of time of learning and vocabulary knowledge on written word learning in children
Source: R Soc Open Sci. 2020 Mar 25;7(3):191597. doi: 10.1098/rsos.191597 (PMC7137956; doi:10.1098/rsos.191597)
Supplement: Mixed effects models [file rsos191597supp2.docx]

**Mixed effects models for each task**

**Table B.1.** *Predictors of semantic categorisation RT performance*

| Fixed effects | | *b* | | SE | | *t* | *p* |
| --- | --- | --- | --- | --- | --- | --- | --- |
| **Intercept** | | **6.98** | | **.03** | | **227.95** | **<.001** |
| Vocab | | -.06 | | .03 | | -1.89 | .064 |
| Delay | | .03 | | .05 | | .63 | .53 |
| **Day** | | **-.19** | | **.02** | | **-9.79** | **<.001** |
| Word type | | .02 | | .01 | | 1.54 | .12 |
| Vocab:Delay | | .11 | | .06 | | 1.73 | .090 |
| Vocab:Day | | -.01 | | .02 | | -.39 | .70 |
| Delay:Day | | .01 | | .04 | | .41 | .68 |
| Vocab:Word type | | -.003 | | .02 | | -.20 | .84 |
| Delay:Word type | | -.01 | | .03 | | -.32 | .75 |
| Day:Word type | | .01 | | .03 | | .51 | .61 |
| Vocab:Delay:Day | | -.01 | | .04 | | -.26 | .80 |
| Vocab:Delay:Word type | | -.02 | | .03 | | -.57 | .57 |
| Vocab:Day:Word type | | -.004 | | .03 | | -.12 | .90 |
| Delay:Day:Word type | | .07 | | .05 | | 1.31 | .19 |
| **Vocab:Delay:Day:Word type** | | **-.12** | | **.06** | | **-2.04** | **.041** |
| Random effects | Variance | | SD | |  |  |  |
| Participant: (intercept) | .03 | | 18 | |  |  |  |
| Participant: Day (slope) | .01 | | .09 | |  |  |  |
| Item: (intercept) | .06 | | .08 | |  |  |  |
| Item: Day (slope) | .001 | | .04 | |  |  |  |

**Table B.2:** *Predictors of speeded recognition RT performance*

| Fixed effects | | *b* | | SE | | *t* | *p* |
| --- | --- | --- | --- | --- | --- | --- | --- |
| **Intercept** | | **6.74** | | **.03** | | **200.48** | **<.001** |
| Vocab | | -.08 | | .04 | | -1.92 | .062 |
| Day | | -.02 | | .03 | | -.70 | .49 |
| Delay | | .07 | | .05 | | 1.50 | .14 |
| Vocab:Day | | -.05 | | .04 | | -1.14 | .26 |
| Delay:Day | | -.07 | | .05 | | -1.55 | .13 |
| Vocab:Delay | | .07 | | .05 | | 1.27 | .21 |
| **Vocab:Day:Delay** | | **.10** | | **.05** | | **2.02** | **.049** |
| Random effects | Variance | | SD | |  |  |  |
| Participant: (intercept) | .02 | | .15 | |  |  |  |
| Participant: Day (slope) | .01 | | .11 | |  |  |  |

**Table B.3:** *Predictors of cued recall accuracy performance*

| Fixed effects | | | *b* | | SE | | *z* | *p* |
| --- | --- | --- | --- | --- | --- | --- | --- | --- |
| Intercept | | | -.11 | | .28 | | -.39 | .69 |
| Vocab | | | .19 | | .25 | | .75 | .45 |
| Day | | | .16 | | .13 | | 1.27 | .20 |
| Delay | | | .26 | | .49 | | .54 | .59 |
| Random effects | Variance | | SD | |  |  |  |  |
| Participant: (intercept) | 2.88 | | 1.70 | |  |  |  |  |
| Item: (intercept) | .57 | | .76 | |  |  |  |  |
| Item: Vocab (slope) | .13 | | .36 | |  |  |  |  |
